# Innovative Combination of Dispersive Solid Phase Extraction Followed by NIR-Detection and Multivariate Data Analysis for Prediction of Total Polyphenolic Content

**DOI:** 10.3390/molecules26164807

**Published:** 2021-08-09

**Authors:** Christoph Kappacher, Markus Neurauter, Matthias Rainer, Günther K. Bonn, Christian W. Huck

**Affiliations:** 1 Institute of Analytical Chemistry and Radiochemistry, Leopold-Franzens University Innsbruck, 6020 Innsbruck, Austria; Christoph.kappacher@uibk.ac.at (C.K.); M.Neurauter@student.uibk.ac.at (M.N.); M.Rainer@uibk.ac.at (M.R.); Guenther.Bonn@uibk.ac.at (G.K.B.); 2 ADSI—Austrian Drug Screening Institute GmbH, 6020 Innsbruck, Austria

**Keywords:** dispersive solid phase extraction, total polyphenolic content, polyvinylpyrrolidone, chemometrics, near-infrared analysis, polyphenol

## Abstract

Recently polyphenols attracted great interest in the field of food and nutrition as well as in the pharmaceutical and cosmetics industries due to their health benefits through antioxidative behavior in the human body. However, because of the high number of compounds characterized as phenols and their structural diversity, quantification of polyphenols turns out to be a highly complex task. Although, a wide variety of analytical methods are used for the determination of total polyphenolic content, they are all found to be lacking in a variety of different tasks, such as their limits of detection and quantification, repeatability, accuracy and specificity. For this reason, a novel approach combining the advantages of solid phase purification, near infrared analysis and multivariate data analysis was investigated for the prediction of total polyphenolic content, suitable for a wide range of sample matrices. Dispersive solid phase extraction was performed and optimized using polyvinylpyrrolidone as sorbent, known to selectively bind polyphenols. Near-infrared detection of adsorbed polyphenols was carried out subsequently. Furthermore, the method was in-house validated, examining selectivity, repeatability and accuracy, working range, as well as multivariate limit of detection and limit of quantification, comparing it with two routinely used methods—namely, Folin–Ciocalteu photometric assay and Löwenthal titration. The novel established method was applied for the prediction of total polyphenolic content in tea and wine samples.

## 1. Introduction

Considering a daily intake of about 1 g per day, polyphenols represent the most abundant antioxidant in human diet, as well as the largest group of phytochemicals, being present in fruit juices, tea, wine, coffee, vegetables, cereals and much more [1,2]. Since the mid-1990s, this compound class gained interest in the field of food, nutrition, pharmaceutical and cosmetics industries due to their ability to reduce oxidative stress caused by excess reactive oxygen species (ROS) associated with cardiovascular diseases, cancers, osteoporosis [2] and an effect said to be for prevention of neurodegenerative diseases and diabetes mellitus [3]. Reasoned by the high number of 8000 known phenolic structures, all of which share an aromatic ring bearing at least one hydroxyl substituent as a structural feature, analysis of this compound class remains challenging [4]. Due to significant variations of their physicochemical properties, chemical complexity and diversity in various matrices, the analysis is commonly performed as sum parameter, expressed as gallic acid or tannic acid equivalent. Although there exist a high number of methods for predicting total polyphenolic content, most of them are found to be lacking in different tasks when it comes to considering their precision and selectivity of analysis, required amount of time or instrumentation for analysis and usage of harmful chemicals. Most of these methods are based on photometric assays or oxidation of phenolic constituents. Two routinely used methods such as Folin–Ciocalteu [5] and Löwenthal titration [6] were chosen as reference methods in order to be able to compare them to the novel established method. Folin–Ciocalteu represents a commonly used spectrophotometric method for the estimation of total polyphenolic content and is based on the reduction of phosphomolybdic-phosphotungstic acid reagent, forming a colored complex in alkaline solutions and in the presence of phenolic compounds, detected at a wavelength of 785 nm. Even though it is routinely used, the method is not very specific and shows wrongly increased results for samples containing reducing agents, including but not exclusively ascorbic acid [2,7]. The Löwenthal titration method arises from apple and cider analysis and relies on the oxidation of polyphenols by potassium permanganate in the presence of indigo carmine, acting as redox indicator. Although chromatographic methods have been widely used for the analysis of phytochemical compounds, the diversity of phenolic compounds and variety of glycosylation and polymerization patterns makes the analysis of total polyphenolic content challenging [2,4]. Mostly, polyphenolic constituents are expressed as a sum parameter such as gallic acid equivalent (GAE) or tannic acid equivalent (TAE) in mg L^−1^.

For this reason, a novel approach based on solid phase purification subsequently followed by near-infrared detection and multivariate data analysis was designed. The aim of this method was to combine the advantages of sample preparation techniques and vibrational spectroscopy. Therefore, it was possible to exclude matrix interference either through selective enrichment of analytes or through chemometric analysis of obtained spectra.

Solid phase purification is commonly applied to the isolation, purification and pre-concentration of analytes in complex sample matrices [8]. A wide variety of sorbents are commercially available, ranging from alkylated silica gels to polymeric residues and inorganic compounds. The use of insoluble polyvinylpyrrolidone (PVP) as a sorbent for the selective removal of plant phenols was published in 1967, proposing a hydrogen bonding between carbonyl functions of PVP and phenols [9]. Using the solid phase residue for enrichment in subsequent analysis, without the need to elute analytes prior to analysis, has so far not been applied. In the current study, the solid resin, including adsorbed polyphenols after extraction, was subsequently analyzed using near-infrared detection in diffuse reflection mode. A calibration set using aqueous gallic acid solutions with varying concentrations was conducted to predict a linear relationship. Extraction was optimized in order to obtain complete adsorption of polyphenols and to create a sufficient analyte-to-sorbent ratio. The next step consisted of in-house validation, examining the approach’s selectivity, repeatability and accuracy and determining its multivariate limit of detection (LOD) and limit of quantification (LOQ), as well as its working range. As a final step, the method was used for the quantification of polyphenols in tea and wine samples in comparison with Folin–Ciocalteu and Löwenthal titration.

## 2. Results

### 2.1. Optimization of Extraction Protocol

HPLC measurements for quantification of remaining gallic acid in sample solution was performed in order to ensure optimal binding conditions for the extraction process. Results for optimization of the pH value for extraction showed a recovery rate of 97.4 ± 0.6% for GA at pH of 3.5, suggesting an almost complete extraction. For pH of 6.0 and 8.0, recovery of 30.3 ± 0.2% and 35.9 ± 0.2%, respectively, could be achieved. The results therefore verify a hydrogen bonding for the interaction between analyte and sorbent. As a next step, extraction time was investigated to ensure fast sample treatment. Therefore, extraction time was varied between 0 min (30 s vortexing) and 40 min while shaking on Thermomix. Recovery rates starting from 96.9 ± 0.2% for 0 s up to 97.2 ± 0.01% could be reached, plateauing after 10 min at approximately 97.2 ± 0.1 and showing declining standard variation with growing extraction time, stopping at 0.01% for 40 min. Extraction time of 20 min was conducted for the following extractions. In order to maximize the analyte-to-sorbent ratio for sensitivity analysis, the loading capacity of PVP was examined. Results for 50 mg PVP showed the lowest recovery rate of 93.7 ± 0.2%, starting to plateau at 97.2% for 100 mg and higher amounts, such as 300 mg and 97.0%. An amount of 100 mg of PVP represented an optimal compromise between adsorption and sensitivity for further NIR analysis. According to Equation (7), a loading capacity of 38.8 mg GA g^−1^ PVP could be achieved using the optimized extraction protocol.

### 2.2. Spectral Interpretation

**Figure 1:**
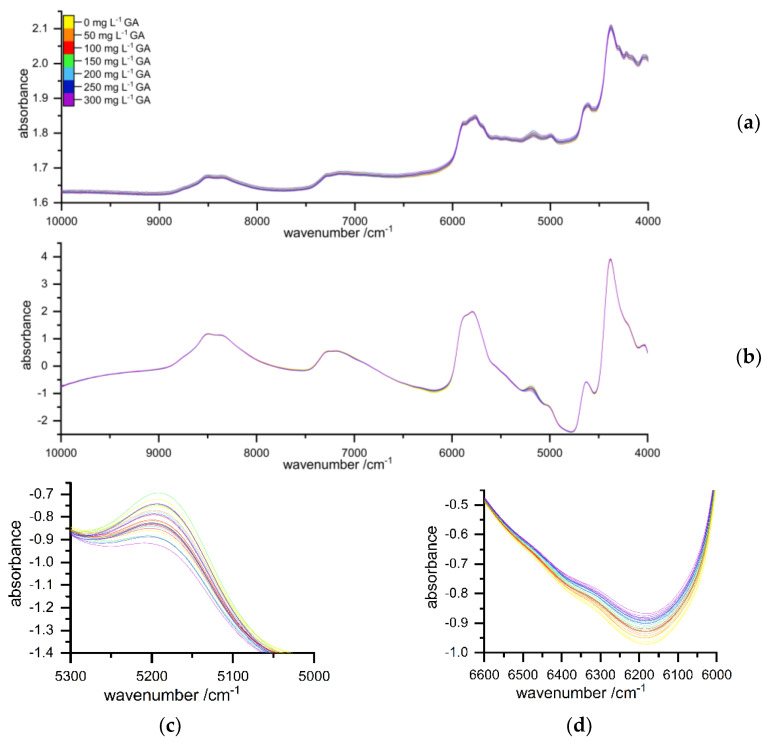
(**a**) Resulting reduced raw spectra for the calibration set between 0 and 300 mg L^−1^ performed according to the optimized extraction protocol. (**b**) Spectra after pretreatment using detrending, reduction, Savitzky–Golay smoothing (polynomial order: 0, smoothing points: 19) and standard normal variate. (**c**) Spectral cutouts of Figure 1b between 5300 and 5000 cm^−1^ depicting vibrations of remaining water. (**d**) Spectral cutouts ranging from 6550 to 6000 cm^−1^ showing highly correlated vibrations according to the gallic acid content.

Significant differences in the NIR spectra from 4350–4400, 4500–4640, 5000–5320, 6000–6550, 6900–7330 and 7360–8040 cm^−1^ were observed. Mentioned spectral regions showed a visually high correlation with concentrations of the standard solution, which is shown representatively in Figure 1d. It concludes that aromatic C-H vibrations were caused by its phenolic basic structure.

Although samples were freeze-dried overnight, aqueous residue could not be prevented. Therefore, the spectral range of 5344 to 4960, presented in Figure 1c, which belongs to OH-stretching vibration of water, was excluded from prediction model building. Additionally, the upper and lower ends of the detected wavelengths, such as 10,000–9612 and 4068–4000, were excluded due to high spectral noise. Resulting wavelength ranges from 9612 to 5344 and 4960 to 4068 were used for model building.

### 2.3. Calibration

**Figure 2:**
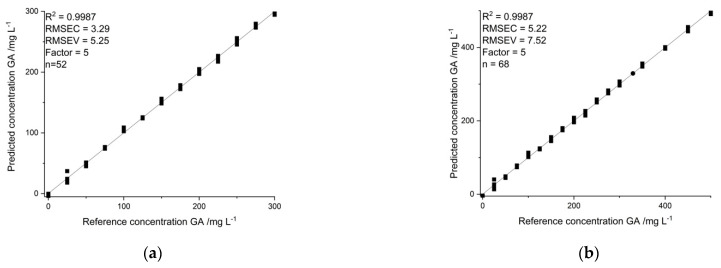
(**a**) Predicted vs. reference plots for calibration ranging from 0 to 300 mg L^−1^ GA. (**b**) Predicted vs. reference plots for calibration ranging from 0 to 500 mg L^−1^ GA. R^2^ = coefficient of determination for calibration, RMSEC = root-mean-square error of calibration; RMSEV = Root-mean-square error of validation; Factor = number of principal components used for calibration; n = number of calibration points.

An optimized extraction protocol was applied to all of the following results. Calibration for NIR analysis was performed four times for a concentration range between 0 and 300 mg L^−1^ GA in steps of 25 mg L^−1^, resulting in 52 calibration points. Multivariate data analysis was performed using partial least squares regression (PLSR). PLSR calibration models based on conducted spectra were fully cross-validated using an NIPALS algorithm. Different component-specific spectral regions as well as full spectral information were investigated for the best prediction performance, derived by comparing calibration parameters such as root-mean-square error of calibration and validation, coefficient of determination and prediction performance for the validation set (Figure 2).

For the F–C reference method, 7-point calibration in duplicate was performed using equidistant standards ranging from 0 to 300 mg L^−1^ GA, resulting in 14 calibration points. Samples followed the same sample preparation and were prepared in triplicate. The second reference method, Löwenthal titration, was calibrated using 5-point calibration ranging from 150 to 350 mg L^−1^ executed in duplicate, as well as a blank that used the indicator in aqueous solution. To ensure adequate polyphenol prediction, a conversion factor into normality of KMnO_4_ was calculated according to Equation (8).

### 2.4. Method Validation

#### 2.4.1. Selectivity

Selectivity of the novel established method was investigated using gallic acid standard solutions spiked with interfering substances such as ascorbic acid, tyrosine and glucose. They were chosen as representatives for vitamins, aromatic amines and sugars, respectively, which could realistically occur in samples. Concentrations of interfering substances were chosen in order to represent either realistic simulations of real-life samples, or to compare results with results reported by Suhui Ma et al. [7]. Glucose was validated using a sample concentration of 100 g L^−1^. Ascorbic acid was added to a final concentration of 1 g L^−1^ and tyrosine to 10 mg L^−1^.

#### 2.4.2. Repeatability and Accuracy

Repeatability for the established method was assessed as relative standard deviation (RSD), whereas accuracy was determined as recovery rate of known concentrations and 95% confidence interval (CI_95_). Therefore 10 samples (*n* = 10) of 150 mg L^−1^ gallic acid standard solutions were prepared and measured within a time interval of 30 min on the same day under the same conditions (materials, equipment) by the same personnel. Recovery rate, relative standard deviation (RSD) and 95% confidence interval (α = 0.05; *Z* = 1.96) were calculated according to Equations (1)–(3).

(1) $$Recovery\;Rate\;\left[\%\right]=\frac{Conc.\;\;supernatant\;\left[\mathrm{mg}\;L^{-1}\right]}{Conc.\;\;standard\;\left[\mathrm{mg}\;L^{-1}\right]}\cdot 100$$

(2) $$RSD=\frac{Standard\;deviation}{Mean}\cdot 100$$

(3) $$CI_{95\%\;}=Mean\;of\;sample\;concentrations\;\pm Z\cdot \frac{Standard\;\;deviation}{\sqrt{n}}$$

#### 2.4.3. LOD and LOQ for PLSR

In the case of multivariate data analysis, there still is no generally accepted calculation for LOD and LOQ, as the instrumental signals are not specific for a particular analyte. Therefore, the approach based on the work of Allegrini and Olivieri was conducted, including the latest IUPAC recommendations: the calculation should be based on the theory of hypothesis testing, as well as including all the different sources of error (calibration and prediction) [10].

(4) $${\mathrm{LOD}}_{\min}=3.3\;[{\mathrm{SEN}}^{-2\;}\mathrm{var}\left(x\right)+h_{0\min}{\;\mathrm{SEN}}^{-2\;}\mathrm{var}\left(x\right)+h_{0\min}\;\mathrm{var}\left(y_{\mathrm{cal}}\right){]}^{1/2}$$

(5) $${\mathrm{LOD}}_{\max}=3.3\;[{\mathrm{SEN}}^{-2\;}\mathrm{var}\left(x\right)+h_{0\max}{\;\mathrm{SEN}}^{-2\;}\mathrm{var}\left(x\right)+h_{0\max}\;\mathrm{var}\left(y_{\mathrm{cal}}\right){]}^{1/2}$$

(6) $${\mathrm{LOQ}}_{\min/\max}=3\cdot {\mathrm{LOD}}_{\min/\max}$$

where SEN is the sensitivity (inverse of the length of regression coefficient), and var(x) represents the variance of the instrument signals. h_0min/max_ is the minimal/maximal distance between a hyperplane for the calibration set and the center of a normalized calibration score space. var(y_cal_) is the variance of calibration concentrations [10].

The limit of detection (LOD) represents the concentration or quantity derived from the smallest measure that can be detected with reasonable certainty for a given analytical procedure, whereas the limit of quantification (LOQ) specifies the lowest concentration at which the analyte cannot only be reliably detected but can also be quantified with an acceptable precision [11].

#### 2.4.4. Working Range

Working range was investigated, applying the optimized extraction protocol and equidistant calibration points in 25 mg L^−1^ steps ranging from 0 to 300 mg L^−1^ GA, four times per concentration. Furthermore, the range of 300 to 500 was conducted in 50 mg L^−1^ steps using four calibration points each. PLSR models using 52 (*n* = 52) and 68 (*n* = 68) points were calculated and results were compared with regard to root-mean-square error of calibration (RMSEC) and validation (RMSEV) and coefficient of determination (R^2^) describing the linear relationship of the data.

#### 2.4.5. Comparison of Novel Established Method to Reference Methods

Working range, values of R^2^, limit of detection and limit of quantification for the novel established PVP extraction method compared with the already established Folin–Ciocalteu and Löwenthal titration are summarized in Table 1 [7]. An optimized extraction protocol was used for the PVP extraction method applied to a concentration range of 0 to 300 mg L^−1^. Additional calibration points from 300 to 500 were conducted for predicting the working range. The following regression parameters could be achieved using 68 calibration points: RMSEC = 5.22, R^2^_cal_ = 0.9987, RMSEV = 7.52, R^2^_val_ = 0.9974 (Factor 5) compared with regression parameters using 52 calibration points: RMSEC = 3.29, R^2^_cal_ = 0.9987, RMSEV = 5.25, R^2^_val_ = 0.9969 (Factor 5). Although the R^2^ for validation was slightly lower for the smaller calibration set, the root-mean-square error of calibration and validation, as well as validation measurements showed significantly better results. Therefore, the following Table 2 and Table 3 represent the results of the smaller calibration set, even though a working range greater than 500 mg L^−1^ could be applied.

The optimized extraction protocol and PLSR model were applied on real-world samples such as red and white wine (Lenz Moser Servus, Burgenland, Austria), as well as black tea (Twinings of London, English Breakfast Tea Medium Loose Tea), all collected at a local market. Red wine was diluted 1:10 due to its expected high polyphenol content, whereas white wine was extracted undiluted. Tea was extracted using 0.5 g and 100 mL deionized water for 1 h under reflux and diluted 1:4. Table 4 shows the results for the prediction of the total polyphenolic content using the three described methods.

Table 4 shows the total polyphenolic content of red and white wine, as well as of extracted black tea for the three mentioned methods. Although the results differ in a large range, a clear trend can be concluded. Lowest results could be observed using the PVP-extraction method. This could be due to a wrongly increased content because of interfering compounds in the sample matrix for F–C and L-C. The PVP-extraction method for white wine shows very low values. Due to the undiluted measurement, the alcohol content of around 11.5% could lead to an ineffective extraction and therefore a lower content of adsorbed polyphenols. Another explanation could be a wrongly in-creased content for F–C and L-C because of the contained amount of sugars and ascorbic acid.

## 3. Discussion

The findings of this study demonstrate that the novel established method is able to predict the total polyphenolic content of samples in various matrices. Validation of the method shows a linear relationship for concentrations from 0 up to 500 mg L^−1^, indicated by a coefficient of determination of 0.999. Although the method was not designed for detection of low concentrations, LOD_min/max_ of 12.3 to 38.4 mg L^−1^ and LOQ_min/max_ of 36.8 to 115.2 mg L^−1^ could be achieved, which enables use of the method for most applications and various matrices. Repeated measurements of 10 samples show high accuracy, represented by 98.7% recovery; nonetheless, its limitations for repeatability are based on a comparatively high relative standard deviation of 10.7%. Superiority in selectivity for two representative interfering substances is shown in Table 3, whereas the novel method shows minimal deviations of less than 2.8% recovery for control measurements, ascorbic acid and tyrosine compared with deviations up to 251% for reference methods. Glucose exhibits the worst result by a recovery rate of 106.9 ± 14.7%, compared with 105.3 ± 0.40% for F–C and 96.4 ± 0.36, which can be explained by its high concentration, as it was left in the solid resin despite a subsequent washing step. Residual glucose in the solid resin caused stickiness of the material, making it hard to ensure uniform particle size for spectroscopic measurement. The presented study shows that the combination of solid phase extraction with subsequent detection enables spectroscopic methods for quantification of low concentrations, and furthermore, it enables quantification of measurements in various matrices. Moreover, it should be pointed out, that this work enables the quantification of polyphenols in various matrices, without restriction of the same sample matrix for calibration and sample measurement.

## 4. Materials and Methods

### 4.1. Chemicals and Samples

Insoluble polyvinylpyrrolidone (PVP), acting as solid phase material, was purchased from SERVA (Heidelberg, Germany) labeled as Polyclar^®^ AT pract. Chemicals used for method establishment were purchased from Sigma-Aldrich (St. Louis, MO, USA), including gallic acid (97.5–102.5%), L-tyrosine (≥98%), D(+)-glucose (≥99.5%) and L-ascorbic acid (99%). For reference analysis, Folin–Ciocalteu phenol reagent (2 M with respect to acid), sodium carbonate (≥99.5%), potassium permanganate (≥99%), sodium oxalate (≥99.5%) were purchased from Sigma-Aldrich (St. Louis, MO, USA). Indigo carmine (≥80% p.a.), used as indicator for Löwenthal titration, was purchased from Carl Roth (Karlsruhe, Germany). HPLC analysis was carried out using methanol (HPLC grade, 99.9%) purchased from VWR Chemicals (Radnor, PA, USA) and diluted trifluoroacetic acid (99%) purchased from Sigma Aldrich (St. Louis, MO, USA).

### 4.2. NIR-Spectral Acquisition Using FT-NIR Spectrometer

NIR spectra of polyphenolic compounds adsorbed to the solid resin were collected in diffuse reflection mode using Büchi NIR-Flex N-500 (Flawil, Switzerland) and a Solids XL top piece. The dried samples were placed in cylindrical quartz cuvettes and placed on the add-on at room temperature (25 ± 1 °C). Spectra were measured in the 10,000 to 4000 cm^−1^ range with a resolution of 8 cm^−1^. Each sample was measured in triplicate, applying 64 scans, rotating the cuvette approximately 120 degrees after each measurement, and an external reference scan after three consecutive samples were carried out. Control of measurements and spectral acquisition were performed using a Büchi NIRWare Operator (Büchi, Flawil, Switzerland).

### 4.3. Spectral Data Treatment

Spectral data processing and interpretation using chemometric tools of recorded spectra were carried out using The Unscrambler × 10.5 Client (CAMO Analytics, Montclair, NJ, USA). First, raw spectra were analyzed using descriptive statistics, and plots were made to identify typical absorbance and spectral regions. As a second step, different pretreatments including standard normal variate (SNV), multiplicative scatter correction (MSC), detrending and first-/second-derivative spectra were computed because of scattering effects induced by non-uniform particle size of the sample. Three consecutive spectra of each sample were averaged after pretreatment. Additionally, Savitzky–Golay smoothing was applied for spectra showing high signal-to-noise ratio, such as SNV, MSC and detrending. Multivariate data analysis was performed using partial least squares regression (PLSR) [12]. The received PLSR model, using the calibration set from 0 to 300 mg L^−1^ GA and 0 to 500 mg L^−1^, respectively, was fully cross-validated.

### 4.4. Dispersive Solid Phase Extraction

**Figure 3:**
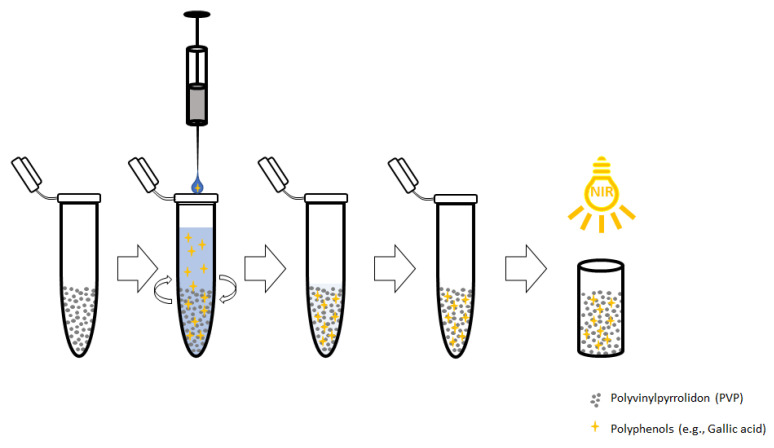
Applied workflow for the PVP extraction method consisting of the addition of PVP as solid resin, adding sample solution, extraction process by shaking, filtration and washing, drying by lyophilization and subsequent near-infrared detection.

Polyvinylpyrrolidone was used in previous research for selective removal of polyphenols in various matrices [13] such as apple juice [14], Nicotiana tabacum [9] and berry plants [15]. The main focus was on the removal of polyphenols as a class of interfering compounds. In this novel approach, PVP was used for the extraction of polyphenols, and subsequent detection and quantification were expressed as gallic acid equivalents. The applied workflow of the dispersive solid phase extraction process is outlined in Figure 3.

A standard stock solution of gallic acid (GA) was prepared and diluted in intentional concentrations in order to generate a calibration set. PVP, acting as extraction material, was weighted in 5 mL Eppendorf tubes. A portion of 4 mL of sample solution was pipetted into prepared Eppendorf tubes and directly vortexed for about 30 s. The tubes were placed in an Eppendorf Thermomix and were allowed to be shaken for a certain amount of time at 25 °C and 1000 rpm. Filter tubes were prepared by adding PE filter frits and were placed in a 12-position SPE manifold system. After complete extraction, the solution was poured into prepared filter tubes, and the liquid phase was removed by suction. The solid supernatant was then placed in a dry freezer and frozen for 1 h at −99 °C. The frozen samples were then dried over night at −92 °C condenser temperature and 50 mbar. Dried samples were ground to a uniform particle size using a spatula in order to generate reproducible spectra. The prepared material was placed in quartz cuvettes and pressed by a metal stamp to avoid air inclusions and uneven particle distribution. Spectra were generated in diffuse reflectance mode.

### 4.5. Method Optimization

To ensure a quantitative and fast analysis, the extraction protocol was optimized for pH level, extraction time and loading capacity of PVP. For this reason, liquid supernatant was collected, and the remaining gallic acid was quantified using high performance liquid chromatography (HPLC) and UV detection. HPLC measurements were carried out using a Shimadzu NextEra containing a Hypersil GOLD C18 column kept at 30 °C and a flow-rate of 1 mL min^−1^. An elution gradient was applied running on 10% methanol and 90% water with 0.1% trifluoroacetic acid for seven minutes, rising to a 50/50 ratio over a two-minute period and returning to initial elution ratio in another two-minute period. An amount of 5 µL was injected per sample, and detection was carried out using a diode array detector set to 276 nm. Calibration was conducted between 2.5 and 25 mg L^−1^ in equidistant intervals of 2.5 mg L^−1^. Measurements were prepared in triplicate, resulting in 30 measuring points for calibration.

As the interaction is mainly based on hydrogen bonding between the carbonyl function of PVP and the hydroxyl function of GA, the pH of the sample solution is supposed to have a huge impact on the recovery of polyphenols [9]. Therefore, pH levels of 3.5, 6 and 8 for 100 mg L^−1^ GA sample solution were conducted, using 6 mol L^−1^ hydrochloric acid and 6 mol L^−1^ sodium hydroxide solution for pH adjustments. Samples in duplicate were prepared using 4 mL of standard solution with varying pH, 100 mg PVP and extraction time of 30 min. Recovery of the extraction was calculated by dividing the obtained concentration value through prepared standard concentration according to Equation (1).

Extraction time was investigated to ensure a fast analysis and complete extraction of PP using 4 mL of sample solution, 150 mg PVP and 200 mg L^−1^ GA standard solution adjusted to pH 3.5. Measurements were performed in duplicate ranging from 0 to 40 min, in 10 min time intervals. Since analyte-to-adsorbent ratio is supposed to have a huge impact on method applications through larger working ranges, loading capacity was evaluated using different amounts of solid phase resin ranging from 50 to 300 mg in 50 mg intervals and 4 mL sample solution of 200 mg L^−1^ GA adjusted to pH 3.5. Adsorption capacity of GA in aqueous solutions was calculated with the optimized extraction protocol according to the following equation:

(7) $$q=\frac{\left(C_{0}-C_{e}\right)\cdot V}{M}$$

where *q* (mg g^−1^) is the amount of total adsorption of GA, *C*_0_ and *C_e_* are initial and equilibrium concentration of GA in solution (mg L^−1^), *V* (L) is the solution volume and *M* (g) is the weighted PVP.

### 4.6. Reference Methods

Folin–Ciocalteu (F–C) and Löwenthal permanganate (L-P) were chosen as reference methods, as they represent two routinely used methods to determine total polyphenolic content [16,17]. The F–C method is based on redox reactions between polyphenols and the F–C reagent through the formation of a blue complex in alkaline solution, read out through a photometric measurement at a wavelength of 765 nm [18]. Although the method is widely applied in food and nutrition science, several compounds such as ascorbic acid, reducing sugars and tyrosine interfere with the F–C reagent and are specified as gallic acid equivalents, wrongly increasing the total polyphenolic content [16]. The F–C method was carried out on real-world samples such as black tea and red and white wine according to Spanos and Wrolstad, with minor modifications. An amount of 50 µL of sample solution was added to a cuvette containing 1.25 mL of 0.2 M F–C reagent, 450 µL deionized water and 1 mL 0.5 M sodium carbonate solution and mixed thoroughly. Cuvettes were placed in a dark container for 2 h at 25 ± 1 °C and measured at 765 nm using a Jenway Genova Plus (Staffordshire, UK) spectrophotometer. For the L-P titration method, total polyphenolic content was determined according to Löwenthal with slight modifications [7]. A 0.002 M KMnO_4_ solution was standardized against sodium oxalate according to Association of Official Agricultural Chemists (AOAC) [18]. Titration was performed using 20 mL of sample solution mixed thoroughly with 5 mL 0.1% indigo carmine indicator in 0.92 M sulfuric acid, experiencing a color change from dark blue to light green and eventually yellow. As the exact stoichiometry is not known, the conversion factor for gallic acid equivalents was determined through the titration of duplicates of 150, 200, 250, 300 and 350 mg L^−1^ and calculated according to the following equation:

(8) $$F=\frac{c_{GA}\cdot V_{s}}{\left(V_{t}-V_{b}\right)\cdot c\cdot t}$$

where *F* is considered as a conversion factor, *c_GA_* is the concentration of the gallic acid standard solution, *V_S_* is the volume of the sample, *V_t_* is the volume of KMnO_4_ solution used for titration of sample, *V_b_* is the volume of KMnO_4_ used for titration of a blank solution, *c* is the concentration of KMnO_4_, and *t* is the titer of KMnO_4_ solution. The total polyphenol content was then calculated according to the rearranged equation using the calculated conversion factor and equal determinants of Equation (8):

(9) $$Polyphenol\;content\;\left[\mathrm{mg}\;L^{-1}\;GAE\right]=\frac{\left(V_{t}-V_{b}\right)\cdot c\cdot t\cdot F}{V_{s}}$$

## 5. Conclusions

The presented method for the prediction of total polyphenolic content using PVP as solid resin with subsequent NIR analysis represents the first method that combines the advantages of solid phase extraction and near-infrared detection. It was shown that adsorbed polyphenols could be quantified without previous elution steps. The novel approach was compared with two routinely used methods and showed its benefits as well as its limitations.

Furthermore, it can be concluded that this method exhibits minimal chemical demand with small amounts of PVP having to be used, which is an environmental and health-harmless substance in comparison with other methods requiring the use of heavy metals, strong oxidizing agents and strong acids and bases [19]. The method further exhibits lower handling times than Löwenthal titration and similar handling times to F–C assay. Automatization of the extraction process, including optimization of the drying process, could be a future extension. This invention could lead the way to further applications of this kind and possibly extend the applicability of near-infrared spectroscopy.

## Figures and Tables

**Table 1:** Analytical curve, working range, coefficient of determination (R^2^), LOD and LOQ of the novel established PVP extraction method, Folin–Ciocalteu (F–C) and Löwenthal titration.

Method	Analytical Curve	Working Range ^a^	R^2^	LOD ^a^	LOQ ^a^
PVP-extr.	y = 0.99987x + 0.1917 ^b^	36.8 to >500	0.999	12.3 to 38.4 ^c^	36.8 to 115 ^c^
F–C ^d^	y = 0.0019x + 0.0096	42.9 to 500	0.999	14.2	42.9
Löwenthal titration ^d^	y = 1256.1x + 48.61	1.47 to 12,000	0.995	0.485	1.47

^a^ mg L^−1^ GAE. ^b^ Regression analysis was performed using standard concentrations in the x axis and measurements in the y axis. ^c^ Calculation of LOD and LOQ was performed according to Equations (4)–(6). ^d^ Data were taken from Suhui Ma et al. [7].

**Table 2:** Repeatability, recovery and 95% confidence interval of the PVP-extraction method, F–C and Löwenthal titration.

Method	Repeatability/% RSD	Recovery/%	CI_95%_
PVP-extr.	10.7	98.7 ± 10.5	138.3 to 157.9 GAE ^a^
F–C ^d^	0.66	102.9 ± 0.21	307.3 to 309.9 GAE ^b^
Löwenthal titration ^d^	0.70	143.5 ± 0.32	7145 to 7207 TAE ^c^

Data expressed as mean ± SEM. ^a^ 150 mg L^−1^ GA standard for *n* = 10 replicates. ^b^ 300 mg L^−1^ GA standard for *n* = 10 replicates. ^c^ 5 g L^−1^ GA standard for *n* = 10 replicates. ^d^ Data were taken from Suhui Ma et al. [7].

**Table 3:** Selectivity of the PVP-extraction method, F–C and Löwenthal titration.

Method	Control	Ascorbic Acid ^c^	Tyrosine ^d^	Glucose ^e^
PVP extr. ^a^	148.1 ± 15.1	149.2 ± 0.07	146.0 ± 0.9	160.4 ± 22.1
PVP extr. ^b^	98.7 ± 10.5	99.4 ± 0.04	97.3 ± 0.6	106.9 ± 14.7
F–C ^a,f^	308.6 ± 0.64	1053 ± 2.4	398 ± 3.3	316 ± 1.2
F–C ^b,f^	102.9 ± 0.21	351.0 ± 0.80	132.7 ± 1.1	105.3 ± 0.40
L-P ^a,f^	7176 ± 16	7803 ± 43	7685 ± 104	6745 ± 25
L-P ^b,f^	102.5 ± 0.23	111.5 ± 0.61	109.8 ± 1.5	96.4 ± 0.36

^a^ Data expressed as mean ± SEM in mg L^−1^. ^b^ Data expressed as mean ± SEM in % recovery. ^c^ 1 g L^−1^ ascorbic acid final concentration in sample. ^d^ 10 mg L^−1^ tyrosine final concentration in sample. ^e^ 100 g L^−1^ glucose final concentration in sample. ^f^ Data were taken from Suhui Ma et al. [7].

**Table 4:** Selectivity of the PVP-extraction method, F–C and Löwenthal titration.

Method	Red Wine	White Wine	Black Tea
PVP-extr.	914 ± 33	39 ± 6	404 ± 30
F–C ^d^	1954 ± 67	261 ± 3	726 ± 24
L-P ^d^	1170 ± 130	246 ± 2	463 ± 24

Data expressed as mean ± SEM in mg L^−1^ GAE. ^d^ Data were taken from Suhui Ma et al. [7].

## Data Availability

Not applicable.

## Sample Availability

Tea and wine samples were all collected at a local market.
